# Preventive Effects of Resveratrol on Endocannabinoid System and Synaptic Protein Modifications in Rat Cerebral Cortex Challenged by Bilateral Common Carotid Artery Occlusion and Reperfusion

**DOI:** 10.3390/ijms19020426

**Published:** 2018-01-31

**Authors:** Gianfranca Carta, Laura Poddighe, Maria Pina Serra, Marianna Boi, Tiziana Melis, Sara Lisai, Elisabetta Murru, Laura Muredda, Maria Collu, Sebastiano Banni, Marina Quartu

**Affiliations:** Department of Biomedical Sciences, University of Cagliari, Cittadella Universitaria, 09042 Monserrato, Italy; giancarta@unica.it (G.C.); laura.poddighe@gmail.com (L.P.); mpserra@unica.it (M.P.S.); marianna.boi@unica.it (M.B.); tizianasilem@yahoo.it (T.M.); saralisy@alice.it (S.L.); m.elisabetta.murru@tiscali.it (E.M.); lauramuredda@gmail.com (L.M.); mcollu@unica.it (M.C.); banni@unica.it (S.B.)

**Keywords:** bilateral common carotid artery occlusion, cerebral cortex, lipoperoxides, endocannabinoid system, PPAR-α, resveratrol, synaptic proteins

## Abstract

This study aims to evaluate the putative roles of a single acute dose of resveratrol (RVT) in preventing cerebral oxidative stress induced by bilateral common carotid artery occlusion, followed by reperfusion (BCCAO/R) and to investigate RVT’s ability to preserve the neuronal structural integrity. Frontal and temporal-occipital cortices were examined in two groups of adult Wistar rats, sham-operated and submitted to BCCAO/R. In both groups, 6 h before surgery, half the rats were gavage-fed with a single dose of RVT (40 mg/per rat in 300 µL of sunflower oil as the vehicle), while the second half received the vehicle alone. In the frontal cortex, RVT pre-treatment prevented the BCCAO/R-induced increase of lipoperoxides, augmented concentrations of palmitoylethanolamide and docosahexaenoic acid, increased relative levels of the cannabinoid receptors type 1 (CB1) and 2 (CB2), and peroxisome-proliferator-activated-receptor (PPAR)-α proteins. Increased expression of CB1/CB2 receptors mirrored that of synaptophysin and post-synaptic density-95 protein. No BCCAO/R-induced changes occurred in the temporal-occipital cortex. Collectively, our results demonstrate that, in the frontal cortex, RVT pre-treatment prevents the BCCAO/R-induced oxidative stress and modulates the endocannabinoid and PPAR-α systems. The increased expression of synaptic structural proteins further suggests the possible efficacy of RVT as a dietary supplement to preserve the nervous tissue metabolism and control the physiological response to the hypoperfusion/reperfusion challenge.

## 1. Introduction

The transient bilateral common carotid artery occlusion followed by reperfusion (BCCAO/R) is a model of transient global hypoperfusion [1] that causes detectable and consistent molecular tissue changes, such as an increase of the lipid peroxidation product malondialdehyde and superoxide dismutase activity [2], a decrease of docosahexaenoic acid (DHA), an increase of oxidative stress and neuroinflammation markers and activation of the endocannabinoid system (ECS) [3,4,5]. 

There is an increasing interest in the use of natural products able to ameliorate the brain damage caused by reperfusion-induced oxidative stress. Resveratrol (RVT) (3,4′,5-trihidroxystilbene) is a natural polyphenolic compound found in different plant species and red wine [6,7], occurring in its glycosylated form, also known as polydatin (5,4′-dihydroxystilbene-3-*O*-β-d-glucopyranoside) [8], and produced naturally by some plants in response to injury or upon attack by pathogens [9]. Among its several beneficial activities, RVT acts as a free radical scavenger, induces the antioxidant pathways, inhibits cyclooxygenase-2 (COX-2), modulates gene expression and regulates cell cycle events [6,10]. Preclinical studies have further shown that RVT displays medicinal properties against chronic inflammation [11,12], ageing [6], cardiovascular and neurological disorders [6,7,13,14,15] and in different experimental models of ischemia [16,17,18]. In the nervous system, RVT has been associated with several neuroprotective effects [19,20,21] among which is the ability to revert synaptic plasticity deficits following acute [22] and chronic cerebral hypoperfusion [23]. Interestingly, it has been shown that, during ischemia, RVT-induced neuroprotection does not occur in peroxisome proliferator-activated receptor-α (PPAR-α) knockout mice and that RVT acts as a PPAR-α agonist [24]. PPAR-α is a ligand-activated transcription factor belonging to the nuclear hormone receptors [25,26,27]. Indeed, all members of the PPAR family function as lipid-sensing receptors and, by means of activation or repression of particular genes, can regulate many crucial physiologic metabolic processes and inflammatory responses of the nervous system [28,29,30], as well as inhibit the expression of many proinflammatory molecules by means of receptor-dependent transrepression [31,32]. As part of its transcriptional activity, PPAR-α also induces the expression of genes implicated in mitochondrial and peroxisomal fatty acid β-oxidation [26,33,34], a biochemical pathway with a key role in long and medium chain-length fatty acid metabolism. In addition, it has been demonstrated that PPAR-α upregulates synaptic function in hippocampal neurons, by transcriptional upregulation of cyclic Adenosine MonoPhosphate (AMP) response element-binding protein (CREB) [35].

It is relevant that the BCCAO/R-induced oxidative stress implicates specific cerebral tissue lipid metabolic changes that involve molecules, such as docosahexaenoic acid (DHA) and selected ECS molecules [4], known to behave as endogenous agonists of PPAR-α [36,37,38,39]. We have previously reported on the possibility to improve the tissue physiological response to the brain hypoperfusion/reperfusion metabolic challenge by in vivo administration of dietary natural compounds such as the *Pistacia lentiscus* L. essential oils [3] and the phytocannabinoid β-caryophyllene (BCP) [5]. In this study, we used the BCCAO/R model of hypoperfusion/reperfusion in the rat to probe the preventive effect of a single acute dose of RVT. With this aim, we chose to examine the forebrain areas that, being directly and selectively irrorated by the internal carotid artery branches, appear to be particularly affected by the BCCAO/R [4], and the temporal-occipital cortex that, being served by the basilar artery branches, can be used as a control. Thus, by means of High Performance Liquid Chromatography-Mass Spectrometry (HPLC-MS) and Western blot analyses, here we examine in the cerebral cortex the fatty acid profile of tissue phospholipids, the tissue concentrations of lipoperoxides, molecules involved in neuroinflammation and indicative of oxidative stress, the receptors CB1, CB2, PPAR-α and their endogenous ligands and the enzyme cyclooxygenase-2 (COX-2) prior to and after induction of BCCAO/R with or without a pre-treatment with an acute dose of RVT. In order to evaluate whether RVT treatment may influence neuroplasticity, as previously suggested [35], we analyzed synaptophysin, syntaxin-3 and post-synaptic density protein-95 (PSD-95) as markers of synaptic plasticity. Results are discussed in view of the possible significance of selected molecules as early markers of an ongoing transient cerebral global hypoperfusion and of the prospect to use RVT as a dietary supplement to control the physiological, pro-inflammatory response to the hypoperfusion/reperfusion challenge. 

## 2. Results

### 2.1. Analysis of Fatty Acid Profiles, Endocannabinoids and Congeners in Brain Tissue

**Figure 1:**
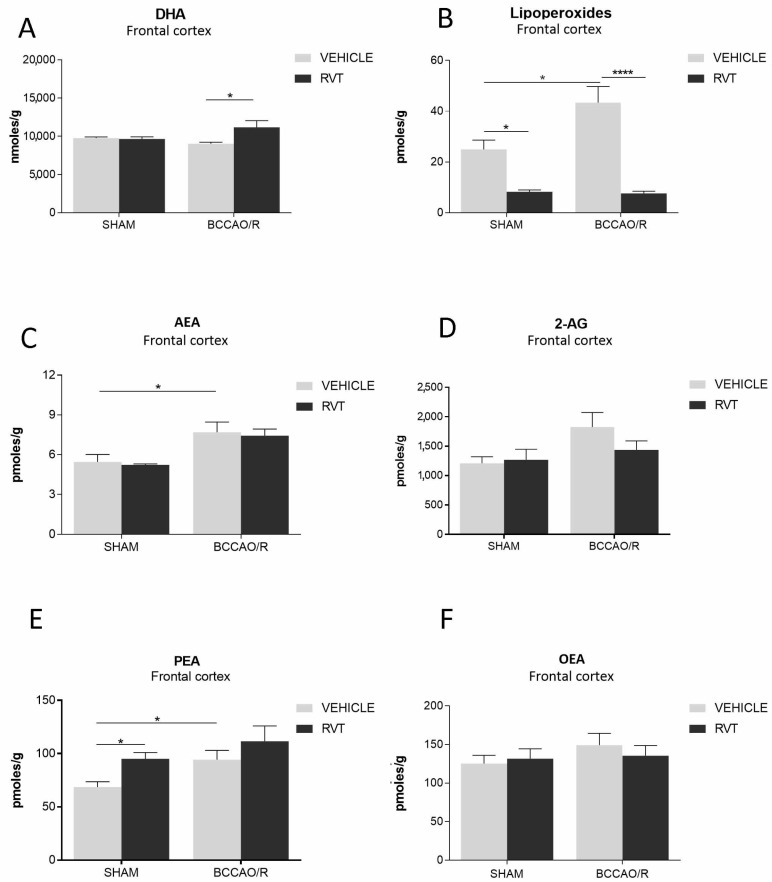
High performance liquid chromatography (HPLC) analysis of frontal cortex of sham-operated and bilateral common carotid artery occlusion followed by reperfusion (BCCAO/R) vehicle-treated and RVT pre-treated rats, respectively. (**A**) Docosahexaenoic acid (DHA), (**B**) lipoperoxides, (**C**) arachidonoyl ethanolamide (AEA), (**D**) 2-arachidonoylglycerol (2-AG) (**E**) palmitoylethanolamide (PEA) and (**F**) oleoylethanolamide (OEA) concentrations are reported as the mean values of 12 vehicle, either sham or BCCAO/R, and 12 RVT pre-treated, either sham or BCCAO/R, rats. Error bars depict the standard error of the mean (S.E.M.). Asterisks denote significant differences. Two-way ANOVA with the Bonferroni’s test for post hoc analyses was applied to evaluate statistical differences between groups. * *p* < 0.05; **** *p* < 0.0001 (see Table 1 for *F*- and *p*-values relevant to the effects of BCCAO/R and RVT pre-treatment and to the interaction between them).

The effects of the administration of RVT on the tissue concentrations of fatty acids, lipoperoxides, endocannabinoids (eCBs) and congeners palmitoylethanolamide (PEA) and oleoylethanolamide (OEA) in the frontal cortex of sham-operated and BCCAO/R rats are summarized in Table 1 and graphically shown in Figure 1. Analysis of the tissue lipid extracts demonstrated that molecular changes following the RVT-pre-treatment were observed only in the frontal cortex (Table 1 and Table 2; Figure 1), whereas no statistically-significant changes were detected in the temporal-occipital cortex. RVT- vs. vehicle-treated differences were detected in the basal concentrations (i.e., in the sham-operated) of lipoperoxides and PEA (Figure 1). In pair-wise comparison of animals in basal conditions, i.e., sham-RVT- vs. sham-vehicle-treated rats, lipoperoxides were decreased by about 66% (post hoc adjusted *p* = 0.0410), while PEA was increased by about 38% (post hoc adjusted *p* = 0.0174) (Table 1 and Table 2; Figure 1). Pair-wise comparisons with Bonferroni’s test revealed that differences also occurred in both groups of BCCAO/R rats; thus, in BCCAO/R-RVT vs. BCCAO/R vehicle-treated animals the concentration of DHA increased (+16%; post hoc adjusted *p* = 0.0174) (Table 1 and Table 2; Figure 1), while that of lipoperoxides decreased (−82%; post hoc adjusted *p* < 0.0001) (Table 1 and Table 2; Figure 1). Accordingly, a significant RVT treatment × BCCAO/R interaction was observed for DHA (F (1, 44): 5.509; *p* = 0.0235) and lipoperoxides (F (1, 39): 5.456; *p* = 0.0247) (Figure 1). The two-way ANOVA followed by Bonferroni’s test also showed an effect of the BCCAO/R per se (Table 1; Figure 1); in fact, in BCCAO/R vs. sham vehicle-treated rats, there was a significant increase of arachidonoylethanolamide (AEA or anandamide) (+41%; *p* = 0.0356), PEA (+39%; *p* = 0.0147) and lipoperoxides concentrations (+74%; *p* = 0.0147). 

### 2.2. Western Blot

**Figure 2:**
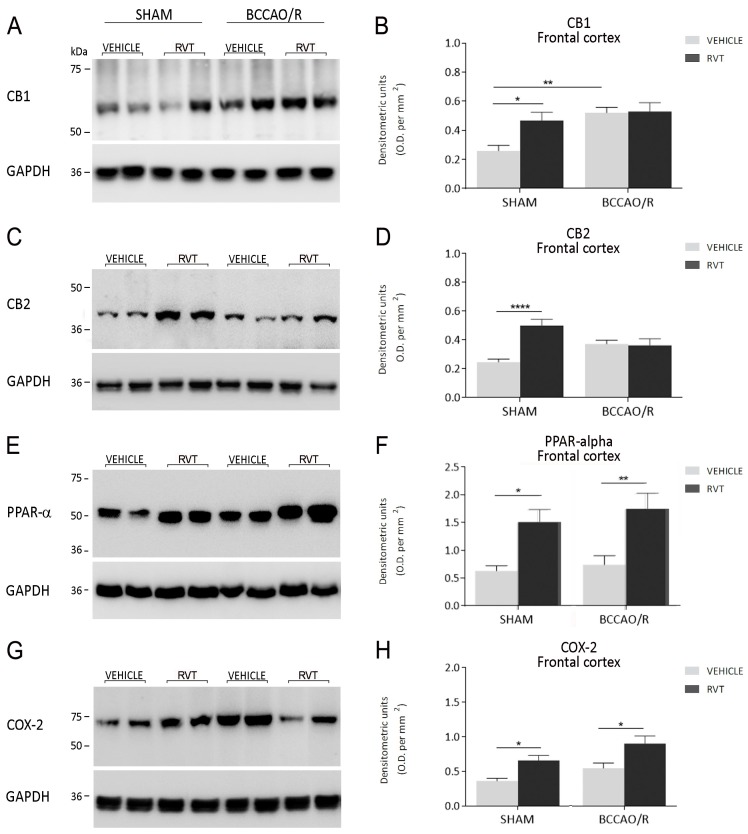
Western blot analysis of cannabinoid receptors CB1 (**A**,**B**) and CB2 (**C**,**D**), peroxisome-proliferator activated receptor-α (PPAR-α) (**E**,**F**) and cyclooxygenase-2 (COX-2) (**G**,**H**) in the frontal cortex of sham-operated and bilateral common carotid artery occlusion followed by reperfusion (BCCAO/R) vehicle-treated and RVT pre-treated rats, respectively. (**B**,**D**,**F**,**H**) densitometric analysis of the band gray levels expressed as a percentage of the optical density (O.D.) ratio of CB1, CB2, PPAR-α- and COX-2-immunostained bands to those of GAPDH. Data are reported as the mean values of 12 sham-operated and 12 BCCAO/R rats and 10 RVT pre-treated, either sham or BCCAO/R, rats. Error bars depict the standard error of the mean (S.E.M.). Asterisks denote significant differences. Two-way ANOVA with the Bonferroni’s test for post hoc analyses was applied to evaluate statistical differences between groups. * *p* < 0.05; ** *p* < 0.01; **** *p* < 0.0001 (see Table 1 for *F*- and *p*-values relevant to effects of BCCAO/R and RVT pre-treatment and to the interaction between them).

**Figure 3:**
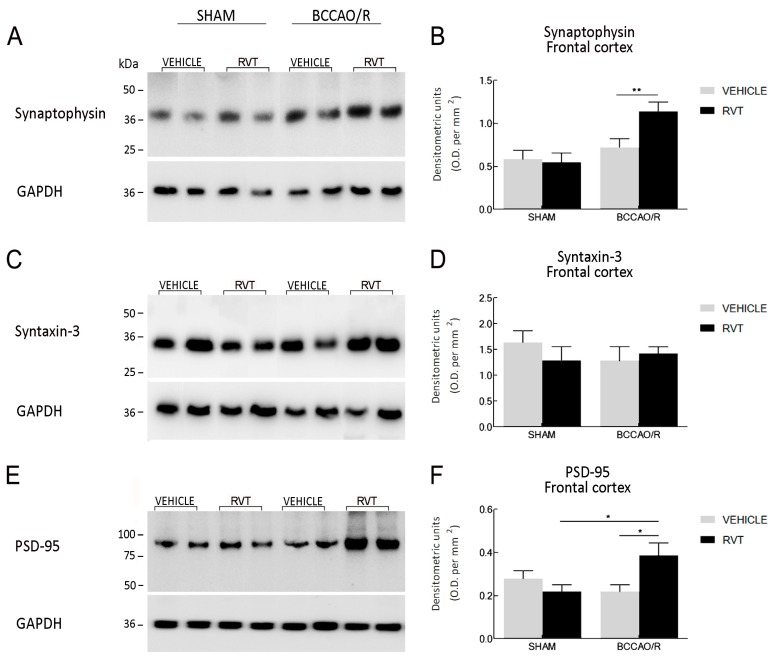
Western blot analysis of synaptophysin (**A**,**B**), syntaxin-3 (**C**,**D**) and post-synaptic density-95 (PSD-95) (**E**,**F**) synaptic proteins in the frontal cortex of sham and bilateral common carotid artery occlusion followed by reperfusion (BCCAO/R) vehicle-treated and RVT pre-treated rats, respectively. (**B**,**D**,**F**) densitometric analysis of the band gray levels expressed as a percentage of the optical density (O.D.) ratio of PPAR-α- and COX-2-immunostained bands to those of GAPDH. Data are reported as the mean values of 12 sham-operated and 12 BCCAO/R rats and 10 RVT pre-treated, either sham-operated or BCCAO/R, rats. Error bars depict the standard error of the mean (S.E.M.). Asterisks denote significant differences. Two-way ANOVA with the Bonferroni’s test for post hoc analyses was applied to evaluate statistical differences between groups. * *p* < 0.05; ** *p* < 0.01 (see Table 1 for *F*- and *p*-values relevant to effects of BCCAO/R and RVT pre-treatment and to the interaction between them).

The effects of the administration of RVT on the relative protein levels of CB1, CB2, PPAR-α, COX-2 and synaptic proteins synaptophysin, syntaxin-3 and PSD-95 are reported in Table 2 and graphically shown in Figure 2 and Figure 3. WB analysis of the tissue homogenates demonstrated that molecular changes following the RVT pre-treatment were observed in the frontal cortex, while no statistically-significant differences were observed in the temporal-occipital cortex. The effect of RVT pre-treatment was evident in the pair-wise comparison of the sham+RVT vs. sham+vehicle rats, with an increase in the relative protein levels of CB1 (+81%; post hoc adjusted *p* = 0.0209), CB2 (+103%; post hoc adjusted *p* < 0.0001), PPAR-α (+139%; post hoc adjusted *p* = 0.0160), COX-2 (+82%; post hoc adjusted *p* 0.0434) and in the pair-wise contrast between the BCCAO/R rats, where PPAR-α (+136%; post hoc adjusted *p* = 0.0066), COX-2 (+65%; post hoc adjusted *p* = 0.0119), synaptophysin (+107% post hoc adjusted *p* = 0.0022) and PSD-95 (+77%; post hoc adjusted *p* = 0.0342) levels significantly increased in BCCAO/R-RVT vs. BCCAO/R vehicle-treated animals (Table 2; Figure 2 and Figure 3). The RVT pre-treatment caused also a general effect on the PSD-95 relative protein levels (*p* = 0.0255), though the pair-wise comparisons did not reach statistical significance (Table 2). The post hoc analysis also showed an effect of the BCCAO/R per se; thus, relative protein levels increased in BCCAO/R vs. sham vehicle-treated rats in the case of CB1 (+101%; post hoc adjusted *p* = 0.0013) (Table 2; Figure 2). Consistently, RVT pre-treatment × BCCAO/R interaction was observed for CB1 (*p* = 0.0414), CB2 (*p* = 0.0006) and synaptophysin (*p* = 0.0414) (Table 2) (Figure 2 and Figure 3). No statistically-significant interaction between the two ANOVA main factors was observed for COX-2 (Table 2).

## 3. Discussion

The main findings of the present study are that, in the frontal cortex, the RVT pre-treatment in a single acute dose exerts significant preventive effects against the BCCAO/R-induced molecular changes, i.e., (a) lowers the lipoperoxide levels in basal conditions and prevents their increase following the BCCAO/R; (b) increases tissue levels of DHA in the BCCAO/R; (c) modulates the activation of the ECS triggered by the BCCAO/R by increasing tissue levels of PEA, CB1 and CB2 in sham animals; (d) induces a general increase of PPAR-α in both sham and BCCAO/R conditions; (e) induces a general increase of COX-2 relative protein levels; (f) increases the SYP and PSD-95 relative protein levels. Present data meet previous observations of the outcome of the transient cerebral hypoperfusion and reperfusion and further support the evidence that the BCCAO/R-induced metabolic changes precede the oxidative stress and neuroinflammation [3,4,40,41].

RVT biological properties have been deeply characterized in both preclinical studies and clinical trials [42]. According to its lipophilic characteristics, RVT is rapidly absorbed and metabolized and, despite its low bioavailability, shows a high efficacy in vivo [42,43]. Studies on RVT pharmacokinetics in rats suggest that its plasma levels peak 5–10 min post-oral administration and that the elimination half-life is 12–15 min [44]. RVT, due to its polyphenolic structure, possesses a low solubility in water (~3 mg/100 mL), according to the definition given by the European Pharmacopeia, with a log p of 3.1 [45]. Several recent studies were focused on novel formulations to improve RVT bioavailability including complexation with solid lipid nanoparticles [46], liposomes [47] and the self-emulsifying delivery system (SEDDS) technique [48]. In studies of SEDDS characterization, it appears that the solubility profile of RVT includes emulsions in edible oils such as olive oil, soybean oil and almond oil [48]. In our experimental model, with no intent to enter the topic of RVT delivery efficiency, we chose as vehicle an edible oil, sunflower oil, whose constituents share a similar fatty acid composition to the rat’s daily diet.

RVT-induced signalosome is complex and contemplates either the activation of intracellular mechanisms [49] or the development of its effects inside the nucleus [42]. Previous studies on the effect of RVT pretreatment in focal cerebral ischemia showed that a single dose of 1 × 10^−6^ and 1 × 10^−7^ g kg^−1^ of RVT significantly reduced the size of cerebral infarction in rats [50] and mice [24]. Our observations demonstrate that RVT acts as neuroprotectant also in the BCCAO/R-induced insult in the frontal cortex, by exerting concomitant anti-oxidative and anti-inflammatory effects, such as the sparing of the DHA, the decrease of lipoperoxides levels and the positive modulation of the anandamide congener PEA. 

At the same time, the RVT-induced modulation of synaptic proteins and the increase of PPAR-α protein levels suggests that, by means of its ability to activate PPAR-α [24], RVT may be also involved in the regulation of synaptic plasticity [35,51]. Interestingly, a lipid-independent role for PPAR-α has been recently reported, by which it may modulate the synaptic function via transcriptional upregulation of CREB [35,52]. Studies have shown that individuals with a DHA-enriched diet are less likely to develop Alzheimer’s disease [53,54]. Indeed, ω-3 fatty acids, such as DHA, are highly enriched in neural membranes and constitute up to 30–40% of the brain phospholipids, where they occur in plasmatic, synaptosomal, microsomal and mitochondrial membranes, as well as in myelin [55]. Since DHA is also a PPAR-α ligand that, in turn, is also capable of upregulating the level of PPAR-α via the PPAR-α signaling itself, it is possible that the maintenance of DHA levels after RVT treatment may contribute to the observed increased levels of synaptic molecules. Such a modulation, on the other hand, might account for structural changes of synaptic elements that, in turn, may also explain the increase of CB receptors and DHA content as components of newly-formed synaptic membranes.

Consistent with our previous findings, in absence of any pre-treatment [3,4], one of the effects through which BCCAO/R can affect the tissue homeostasis is the decrease of DHA tissue content. DHA regulates the efficiency of membrane-dependent molecular mechanisms [56] and is avidly retained in the brain [57,58]. DHA, though, is known to be particularly predisposed to lipid peroxidation [57] and, accordingly, potentially apt at contributing to hypoperfusion-/reperfusion-induced oxidative stress. Interestingly, the pre-treatment with RVT totally prevented the BCCAO/R-induced decrease of DHA. The observation that RVT pre-treatment also induces a rise in the relative protein levels of PPAR-α and COX-2 and a decrease of lipoperoxides suggests that RVT activates multiple and concurrent factors to counteract the BCCAO/R-induced inflammatory reaction, hence helping to preserve the brain structure. Indeed, there is evidence that DHA activates PPAR-α [36,38] and also inhibits COX-2 and prostaglandins formation during neuroinflammation [59]. In this context, it is relevant that DHA is the precursor of local anti-inflammatory autacoids, designated with the generic term of “docosanoids”, that are produced on demand and have been shown to act as negative regulators of inflammation processes otherwise becoming pathological [60]. Recent prophylactic and therapeutic approaches for cerebrovascular disease take into account the pathways of brain accretion and delivery of DHA [56,61,62].

As a matter of fact, in the present study, RVT treatment further triggers a marked decrease of tissue concentrations of lipoperoxides, which are quite unstable compounds capable of extending the free radical oxidative damage and form pro-inflammatory substances [63,64]. Lipoperoxides have been shown to represent one of the hallmarks of BCCAO/R in both cerebral tissue and plasma, leading to the suggestion that they may represent an early marker of the cerebral hypoperfusion-/reperfusion-induced oxidative stress [4]. Several physiopathological conditions, not necessarily associated with early obvious neurological signs [65], share the occurrence of cerebral hypoperfusion episodes for which the detection of molecular indicators in the early hours may be useful in clinical settings to prevent irreversible cerebral damage. Interestingly, the present data are also consistent with previous results obtained in serum from patients undergoing carotid endarterectomy, where increased levels of lipoperoxides and isoprostanes and a concomitant increase of their catabolism in peroxisomes have been shown to be directly correlated with the hypoperfusion/reperfusion-induced oxidative challenge [66]. In particular, it is interesting that peroxisomal β-oxidation increased during the first 30 min of reperfusion only in patients having contralateral carotid stenosis higher than 50% [66]. 

As for the COX-2 expression, its increase after RVT administration may be explained by the degradation of eicosanoids. In fact, it is known that COX-2 can be partially degraded through free radical chain reaction triggered by its same reaction products, i.e., the eicosanoids [67]. A lower level of eicosanoids may therefore sustain COX-2 levels.

We have previously demonstrated that the BCCAO/R model of transient global hypoperfusion/reperfusion activates the ECS and can lead to the accumulation of eCBs and PEA [4,5]. PEA participates in the endogenous defense against neuroinflammation [68,69,70,71]. Levels of PEA have been shown to increase in stroke patients during the acute ischemic phase in which they may play a neuroprotective role through multiple potential mechanisms [72]. Exogenous administration of PEA in an acute stroke model is effective in reducing the infarct size [73]. Our data indicate that, after a single dose of RVT, the tissue levels of PEA increase in the sham-operated rats, while they do not change after induction of BCCAO/R. Importantly, PEA exerts its neuroprotective effects by activating PPAR-α [31,37,39] and inhibiting COX-2 activity [69]. Interestingly, it has been recently shown that the exogenous administration of a co-micronized PEA + polydatin formulation, given orally, resulted in proficient anti-inflammatory and anti-oxidant effects [74]. It has to be considered that, as part of its transcriptional activity, PPAR-α also induces the expression of genes implicated in mitochondrial and peroxisomal fatty acid β-oxidation [26,33,61]. Peroxisomes have a crucial role in the metabolism of reactive oxygen species and lipids, and their importance in brain physiopathology is well established. We hypothesize that, in our model, RVT and PEA, both agonists of the PPAR-α receptor, may act synergistically at it thereby increasing peroxisome activity (such as the β-oxidation) to enhance the degradation of eicosanoids and, generally, to counteract the oxidative stress induced by the BCCAO/R. According to the possibility of a synergistic effect of RVT and PEA, it has been shown that PEA, similarly to RVT, upon interaction with PPAR-α may work against the increase of lipid peroxidation, protein nitration, expression of key proinflammatory enzymes, such as inducible nitric oxide synthase (iNOS) and COX-2, and production of reactive oxygen species [31,74]. 

Interestingly, it has been shown that induction of the anti-inflammatory nuclear receptor PPAR-α elicits an increase of PEA and OEA [75]. As for PEA, this finding is in agreement with the key role of endogenous PEA that, acting at PPAR-α, can provide a no-go signal that hinders the development of acute inflammation [39,76]. Thus, in the presence of exogenous RVT, the tissue challenged by the BCCAO/R vs. control tissue shows an unchanged AEA level and an increase of PEA concentration; possibly, the last one appears to be the main contributor to the modulation of the endogenous anti-inflammatory tone. Surprisingly, we did not detect any change in OEA levels. In fact, if the increase of PEA is related to PPAR-α activation, one may have expected to find increased OEA levels, as well. A possible explanation of this discrepancy may lie in the activity of the different degrading enzymes for PEA and OEA, namely *N*-acylethanolamine hydrolyzing acid amidase (NAAA) and fatty acid amide hydrolase (FAAH), respectively [77,78]. It has been suggested that amplifying the endogenous PEA activity at PPAR-α through inhibition or modulation of its enzymatic breakdown may represent a complementary therapeutic approach to counteract neuroinflammation [71,79,80]. However, while the design of specific inhibitors has contributed to demonstrate the role of NAAH in the termination of PEA signaling [76], this role is not as well consolidated for FAAH and has been shown to depend on a specific tissue context [76,81,82]. Therefore, future studies will certainly aim at evaluating a possible role of NAAA and FAAH activities during the hypoperfusion/reperfusion induced by the BCCAO/R. 

In our basic experimental setting, it is not feasible to speculate whether the increased levels of PPAR-α receptor protein are accompanied by an increased activity induced by its ligands, such as PEA, DHA and the RVT itself, or vice versa. However, altogether, the increase of PEA concentration associated with the increase of PPAR-α protein expression, the marked decrease of lipoperoxides and parallel increase of DHA in the BCCAO/R+RVT rats are elements of a complex picture that suggests an augmented endogenous anti-inflammatory response to BCCAO/R-induced oxidative stress. 

## 4. Materials and Methods

### 4.1. Animals and Keeping

For 1 week before the experiment set off, male Wistar rats (Harlan-Italy, Udine, Italy), weighing 210 ± 20 g (mean ± SD), were housed under controlled temperature (21 ± 2 °C), relative humidity (60 ± 5%) and an artificial 12 h light/dark cycle, avoiding all stressful stimuli. Animal handling and care throughout the experimental procedures met with national (Legislative Decree n. 26, 04/04/2014) and international (Directive 2010/63/EU in Europe) laws and policies. The experimental protocols were carried out in compliance with the guidelines of the Animal Ethics Committee of the University of Cagliari (approval code No. 06/2013, 05/31/2013). Standard laboratory food (A04, Safe, Augy, France) and water were freely available ad libitum. 

According to the optimum standard for the evaluation of lipids in tissue and plasma, animals received no food for 12 h before surgery. 

Rats (*n* = 88) were randomly assigned to 2 groups that received a pre-treatment 6 h before the surgery: one group (vehicle-treated) was pre-treated with the vehicle, i.e., 0.3 mL of sunflower oil, while the other group (RVT-treated) was previously gavage-fed (with the help of a feeding needle) with 40 mg of RVT (Tokyo Chemical Industry Co., (TCI America), Portland, OR, USA) (corresponding to 180 mg·kg^−1^), dissolved in 0.3 mL of sunflower oil as the vehicle. The choice to use a vehicle was made to guarantee that an amount of RVT as accurate as possible can be administered via gavage and to facilitate the absorption of RVT. Each group was further subdivided into sham-operated or submitted to BCCAO/R.

### 4.2. Surgery

The surgical procedure for induction of BCCAO/R was adapted from the method of Iwasaki et al. [83] and performed in all cases between 13:00 and 16:30 p.m. Rats were anesthetized with intraperitoneal administration of Equithesin (4.2% *w/v* chloral hydrate, 2.12% *w/v* MgSO4, 16.2% *w/w* pentobarbital, 39.6% *w/w* propylene glycol and 10% *w/w* ethanol in sterile distilled H_2_O) (5 mL/100 g bodyweight). After a midline cervical incision and blunt dissection of muscles, the common carotid arteries (CCA) were exposed taking care to leave the vagus nerve intact. Cerebral blood flow reduction was produced by placement of two atraumatic microvascular clips for 30 min on CCA. The reperfusion period was achieved by removing the clips and restoring blood flow through the stenosed vessels for 60 min. The control animals, used to determine the effects of anesthesia and surgical manipulation on the results, were represented by sham-operated rats that underwent surgery without CCA occlusion.

### 4.3. Sampling

At the end of the procedure, brain samples were collected as fresh tissue for lipid analysis and Western blot. The frontal cortex was rapidly dissected out by a transverse cut made at the level of the optic chiasm, at the approximate bregma level of −1.0 mm [84] and frozen at −80 °C until HPLC or Western blot analysis. Temporal-occipital cortex, dissected out by a transverse cut at the approximate bregma level of −4.5 mm, was also sampled as a control cortical area not irrorated by the internal carotid artery branches. For each assay, the investigator was blind with respect to the experimental condition of rats.

### 4.4. Measurement of Fatty Acid Composition of Tissue Phospholipids

Total lipids were extracted from different brain areas using chloroform/methanol 2:1 (*v/v*) containing 2 μg of vitamin E. Aliquots were mildly saponified as previously described [85] in order to obtain free fatty acids for high-performance liquid chromatography (HPLC) analysis. Separation of fatty acids was carried out with an Agilent 1100 HPLC system (Agilent Technologies) equipped with a diode array detector as previously reported [86]. Concentrations (nmoles/g; nmoles/mL) are shown as histograms in Figure 1.

### 4.5. Endocannabinoid and Congener Quantification

Frozen tissues from 24 vehicle-treated and 24 RVT pre-treated adult rats were homogenized and extracted with a chloroform-methanol solution (2:1, *v/v*), containing internal deuterated standards for anandamide (AEA), 2-arachidonoyl-monoacylglycerol (2-AG), palmitoylethanolamide (PEA) and oleoylethanolamide (OEA), quantification by isotope dilution ([2H]^8^ AEA, [2H]^5^ 2-AG, [2H]^4^ PEA, [2H]^4^ OEA; Cayman Chemical, Ann Arbor, MI, USA). AEA, 2-AG, PEA and OEA were quantified by liquid chromatography-atmospheric pressure chemical ionization-mass spectrometry (1100 HPLC system (Agilent Technologies, Santa Clara, CA, USA) equipped with an MS Detector 6110 single quadrupole) and using selected ion monitoring at M1 values for the four compounds and their deuterated homologs, as described previously [87]. Concentrations (nmoles/g; nmoles/mL) are shown as histograms in Figure 1. 

### 4.6. Western Blot

Tissue homogenates from 24 vehicle-treated and 20 RVT pre-treated adult rats were prepared in a 2% solution of sodium dodecyl sulfate (SDS) containing a cocktail of protease inhibitors (cOmplete, Mini Protease Inhibitor Cocktail Tablets, Roche, Basel, Switzerland). Protein concentrations were determined using the Lowry method of protein assay [88] with bovine serum albumin as the standard. Proteins for each tissue homogenate (40 μg), diluted 3:1 in 4× loading buffer (NuPAGE LDS Sample Buffer 4×, Novex by Life Technologies, Carlsbad, CA, USA), were heated to 95 °C for 7 min and separated by SDS-polyacrylamide gel electrophoresis (SDS-PAGE) using precast polyacrylamide gradient gel (NuPAGE 4–12% Bis-Tris Gel Midi, Novex by Life Technologies) in the XCell4 Sure Lock^TM^ Midi-Cell chamber (Life Technologies). Internal mw standards (Precision Plus Protein^TM^ WesternC^TM^ Standards, Bio-Rad, Hercules, CA, USA) were run in parallel. Two gels at a time were run for Coomassie staining and immunoblotting, respectively. Proteins for immunoblotting were electrophoretically transferred on a polyvinylidene fluoride membrane (Amersham Hybond^TM^-P, GE Healthcare, Little Chalfont, U.K.) using the Criterion^TM^ Blotter (Bio-Rad). Blots were blocked by immersion in 20 mM Tris base and 137 mM sodium chloride (TBS) containing 5% milk powder and 0.1% Tween 20 (TBS-T), for 60 min at room temperature and incubated overnight at 4 °C with rabbit polyclonal antisera directed against CB1 receptor (Synaptic System, Göttingen, Germany), diluted 1:500, CB2 receptor (Cayman Chemical, Ann Arbor, MI, USA), diluted 1:1000, PPAR-α (Thermo Scientific, Waltham, MA, USA), diluted 1:1000, COX-2 (residues 570–598) (Cayman Chemical), diluted 1:200, synaptophysin (Cell Signalling Technology, Leiden, The Netherlands), diluted 1:1000, syntaxin-3 (AbCam), diluted 1:500, and mouse polyclonal antiserum against PSD-95 (Thermo Scientific), diluted 1:1000 in TBS containing 5% milk powder and 0.02% sodium azide, used as primary antisera. After TBS-T rinse, blots were incubated for 60 min, at room temperature, with peroxidase-conjugated goat anti-rabbit serum (Sigma Aldrich, Milan, Italy), diluted 1:10,000 in TBS-T. Loading controls were obtained by stripping and immunostaining the membranes with a mouse monoclonal antibody against the housekeeping protein glyceraldehyde 3-phosphate dehydrogenase (GAPDH) (EMD Millipore, Darmstadt, Germany), diluted 1:1000, as primary antiserum, and a peroxidase-conjugated goat anti-mouse serum (EMD Millipore-Merck, Darmstad, Germany), diluted 1:5000, as secondary antiserum. In order to control for non-specific staining, blots were stripped and incubated with the relevant secondary antiserum. After TBS-T rinse, protein bands were visualized using the ECL chemiluminescent system according to the protocol provided by the company (GE Healthcare), under ImageQuant LAS 4000. Approximate molecular weight (mw) and relative optical density (O.D.) of immunolabeled protein bands were evaluated by a “blind” examiner and were quantified by comparing the position of relevant bands on the digital images with those of the GAPDH bands, respectively. The ratio of the intensity of CB1-, CB2- PPAR-α-, COX-2-, synaptophysin-, syntaxin-3- and PSD-95-positive bands to the intensity of GAPDH-positive ones was used to compare relative expression levels of these proteins following BCCAO/R procedure. The O.D. was quantified by Image Studio Lite Software (Version 5.2, Li-Cor, http://www.licor.com/bio/products/software/image_studio_lite/) and is shown as histograms in Figure 2 and Figure 3.

### 4.7. Statistical Analysis

Data from the four experimental subgroups, i.e., vehicle- and RVT-treated sham-operated animals, and vehicle- and RVT-treated BCCAO/R rats, are depicted in Figure 1, Figure 2 and Figure 3 as the mean ± standard deviation (S.D.). Two-way analysis of variance (ANOVA) (main factors: (a) RVT-treatment (i.e., vehicle- vs. RVT-treatment) and (b) BCCAO/R (i.e., sham-operation vs. BCCAO/R) was performed using GraphPad Prism 7.03 for Windows (GraphPad Software, La Jolla CA, USA, www.graphpad.com). Wherever appropriate (i.e., *p* for the main factors and their interaction <0.05), multiple pair-wise contrasts were made, and the multiplicity adjusted *p*-value for each comparison was calculated using Bonferroni’s post hoc test.

## 5. Conclusions

The present study showed that RVT pre-treatment prevents the BCCAO/R-induced increase of pro-inflammatory molecules supporting the concept that multiple mechanisms may cope with the molecular dysregulation induced by BCCAO/R and indicate that it may be an excellent therapeutic agent to preserve tissue metabolism and prevent the upshots of the hypoperfusion/reperfusion challenge.

## Figures and Tables

**Table 1:** Frontal cortex. Quantitative data (mean, S.E.M. (standard error of the mean)) of docosahexaenoic acid (DHA), lipoperoxides, endocannabinoids 2-arachidonoylglycerol (2-AG) and arachidonoylethanolamide (AEA), and endocannabinoid congeners palmitoylethanolamide (PEA) and oleoylethanolamide (OEA) obtained after resveratrol (RVT) pre-treatment by means of High Performance Liquid Chromatography-Mass Spectrometry (HPLC-MS).

Marker		Experimental Groups			
		SHAM-VEHICLE	BCCAO/R-VEHICLE	SHAM-RVT	BCCAO/R-RVT
		Number of Samples			
		12	12	8	8
		**Quantitative Data**			
DHA	Mean	9772	9041	9659	11186
	S.E.M.	156.80	206.60	360.80	1104
Lipoperoxides	Mean	24.91	43.37	7.785	7.696
	S.E.M.	3,731	6.338	0.858	0.910
2-AG	Mean	1210	1827	1267	1425
	S.E.M.	110.20	245.10	204.50	184.50
AEA	Mean	5.45	7.696	5.232	7.483
	S.E.M.	0.567	0.769	0.0825	0.517
PEA	Mean	68.50	94.36	95.56	111.70
	S.E.M.	5.112	8.584	2.103	5.737
OEA	Mean	125.30	149.20	131.50	133.30
	S.E.M.	10.60	15.21	4.572	4.658

Legend: BCCAO/R, bilateral common carotid artery occlusion/reperfusion.

**Table 2:** Frontal cortex. *F*-values and significance levels from two-way ANOVA performed on data obtained after BCCAO/R and resveratrol (RVT) pre-treatment by means of HPLC-MS and Western blot.

Method	Marker	ANOVA Factors						
		BCCAO/R		RVT Treatment		RVT Treatment × BCCAO/R		
		*F*-Value	*p*-Value	*F*-Value	*p*-Value	*F*-Value	*p*-Value	DF
HPLC-MS	DHA (22:6)	0.6823	ns	4.462	0.0404	5.509	0.0235	1, 44
	Lipoperoxides	4.783	0.0348	41.17	<0.0001	5.456	0.0247	1, 39
	AEA	15.08	0.0004	0.1696	ns	0.01295	ns	1, 40
	2-AG	4.629	0.0375	0.8341	ns	1.513	ns	1, 40
	PEA	12.71	0.0010	13.63	0.0007	0.6348	ns	1, 40
	OEA	1.735	ns	0.1256	ns	0.908	ns	1, 40
Western blot	CB1 receptor	11.45	0.0016	5.195	0.0203	4.44	0.0414	1, 40
	CB2 receptor	0.02838	ns	12	0.0014	14.07	0.0006	1, 37
	PPAR-α	0.7778	ns	22.08	<0.0001	0.1082	ns	1, 38
	COX-2	8.007	0.0073	18.88	<0.0001	0.1504	ns	1, 39
	Synaptophysin	3.198	ns	11.63	0.0016	4.456	0.0414	1, 38
	PSD-95	0.00221	ns	5.457	0.0255	1.485	ns	1, 34
	Syntaxin-3	0.4759	ns	0.1772	ns	1.473	ns	1, 35

COX-2, cyclooxygenase-2; DF, degrees of freedom; ns: not significant; PPAR-α, peroxisome-proliferator activated receptor-α; PSD-95, post-synaptic density protein 95.

## Abbreviations

AEA	arachidonoyl ethanolamide or anandamide
2-AG	2-arachidonoylglycerol
BCCAO/R	bilateral common carotid artery occlusion followed by reperfusion
BCP	β-caryophyllene
CB	cannabinoid receptor
COX-2	cyclooxygenase-2
DHA	docosahexaenoic acid
eCBs	endocannabinoids
ECS	endocannabinoid system
FAAH	fatty acid amide hydrolase
NAAH	*N*-acylethanolamine hydrolyzing acid amidase
OEA	oleoylethanolamide
PEA	palmitoylethanolamide
PPAR-α	peroxisome-proliferator activated receptor-α
PSD-95	post-synaptic density protein-95
RVT	resveratrol
